# Plasma Metabolic Outliers Identified in Estonian Human Knockouts

**DOI:** 10.3390/metabo15050323

**Published:** 2025-05-13

**Authors:** Ketian Yu, Estonian Biobank Research Team, Karol Estrada, Tõnu Esko, Mart Kals, Tiit Nikopensius, Jaanika Kronberg, Urmo Võsa, Arthur Wuster, Lorenzo Bomba

**Affiliations:** 1 Genomics, BioMarin Pharmaceutical, Novato, CA 94949, USAarthur.wuster@bmrn.com (A.W.); 2 Estonian Genome Centre, Institute of Genomics, University of Tartu, 51010 Tartu, Estoniajaanika.kronberg@ut.ee (J.K.);

**Keywords:** human knockouts, metabolomics, predicted loss of function, pyrimidine pathway, pharmacogenomics, drug development

## Abstract

**Background/Objectives:** Metabolomics, in combination with genetic data, is a powerful approach to study the biochemical consequences of genetic variation. We assessed the impact of human gene knockouts (KOs) on the metabolite levels of Estonia Biobank (EstBB) participants and integrated the results with electronic health record data. **Methods:** In 150,000 EstBB genotyped participants, we identified 723 KOs with 152 different predicted loss of function (pLoF) variants in 115 genes. For those KOs and 258 controls, 1387 metabolites were profiled using ultra-high-performance liquid chromatography–tandem mass spectrometry. **Results:** We identified 48 associations linking rare pLoF variants in 22 genes to 43 metabolites. Out of 48 associations, 27 (56%) were found in genes that cause inborn errors of metabolism. The top associations identified in our analysis included genes and metabolites involved in the degradation pathway of the pyrimidine bases uracil and thymine (*DPYD* and *UPB1*). We found *DPYD* gene KOs to be associated with elevated levels of Uracil, confirming that DPD-deficiency is a leading cause of severe 5-Fluorouracil toxicity. Overall, 54% of reported associations are gene targets of approved drugs or bioactive drug-like compounds. **Conclusions:** Our findings contribute to assessing the impact of human KOs on metabolite levels and offer insights into gene functions, disease mechanism, and drug target validation.

## 1. Introduction

Metabolomics is a robust method for discovering biomarkers and, when combined with genetic data, for determining the biochemical consequences of genetic variation. Genome-wide association studies (GWAS) have identified metabolic quantitative trait loci (mQTLs) that affect metabolite levels, providing insights into gene function and disease mechanisms [1]. Moreover, mQTLs aid the identification and validation of potential drug targets and are enriched near genes of pharmacological interest [2]. While mQTLs are typically common genetic variants, the focus has recently shifted towards the rare spectrum of genetic variation with larger effects on metabolites levels [3,4,5,6,7]. Studying rare variants with a predicted severe impact on protein function can inform drug discovery efforts assisting with the interpretation of the phenotypic consequences of partial or complete gene “knockouts” (KOs) in humans. Metabolite levels of KOs for genes encoding a drug target may mimic the pharmacological modulation of a drug, providing an “experiment of nature” to inform drug development [8]. To expand this concept, we leveraged 723 individuals with predicted loss of function (pLoF) variants from the Estonian Biobank (EstBB) with 1387 metabolites profiled using ultra-high-performance liquid chromatography–tandem mass spectrometry. The Estonian population is enriched with human knockout variants due to its population history and structure, characterized by high homozygosity resulting from recent and regional bottlenecks. Therefore, the unique genetic basis of the Estonian population provides a strong rationale and motivation for this study. We define a human knockout as an individual carrying at least two predicted homozygous pLoF variants in a gene. The pLoF variants were validated using Sanger sequencing. We developed a method to identify metabolic outliers and integrate these results with electronic health record (EHR) data.

## 2. Materials and Methods

### 2.1. Study Description

In this study, we investigated the relationship between metabolite levels and rare genetic variation in the EstBB. The EstBB is a volunteer-based biobank which currently contains more than 210,000 participants aged 18 years and older, closely reflecting the age, sex, and geographical distribution of the adult Estonian population [9]. The participants provided demographic and epidemiological information through a questionnaire. Data from national registries, including EHR are also regularly linked with biobank participants in International Classification of Diseases, 10th Revision (ICD-10) coding.

Venous blood samples were drawn for DNA, white blood cells, and plasma tests. The EstBB samples were genotyped at the Core Genotyping Lab of the Institute of Genomics, University of Tartu, using Illumina genotype arrays (Global Screening Arrays v1.0, v2.0, and v2.0 array with Estonian customization). Individuals whose genetic sex, determined by X chromosome heterozygosity, did not match their recorded sex in the phenotype data were excluded from the analysis. Prior to imputation, variants were filtered based on call rate (<95%), deviation from Hardy–Weinberg equilibrium (*p* < 1 × 10^−4^ for autosomal variants), and minor allele frequency (<1%). Pre-phasing was conducted using Eagle v2.3 [10], and imputation was performed with Beagle [11], using an Estonian-specific reference panel of 2297 Estonian whole-genome sequencing samples [12].

Array and imputed genotype data were available for a total of 152,357 EstBB participants. The majority (~95%) of pLoF variants were derived from array data. Additional variant selection was based on high-quality imputed data (INFO score > 0.5).

### 2.2. Variant Annotation and Gene Selection

All variants were annotated using the Variant Effect Predictor (VEP, version 94) [13] and the Loss-Of-Function Transcript Effect Estimator (LOFTEE, version 1.0) plugin [14].

Our analyses focused on Estonian knockout genes identified across the genome and selected disease genes with heterozygous carriers of pathogenic and pLoF variants, employing two different strategies for gene selection.

Strategy 1: We selected high confidence (HC) pLoF variants with a minor allele frequency (MAF) < 2%, ensuring there were at least two complete KOs (homozygous carriers of the risk allele) within a gene. We then filtered out genes that were not reported in the Online Mendelian Inheritance in Man (OMIM) [15] database or the Open Targets Platform [16].

**Figure 1:**
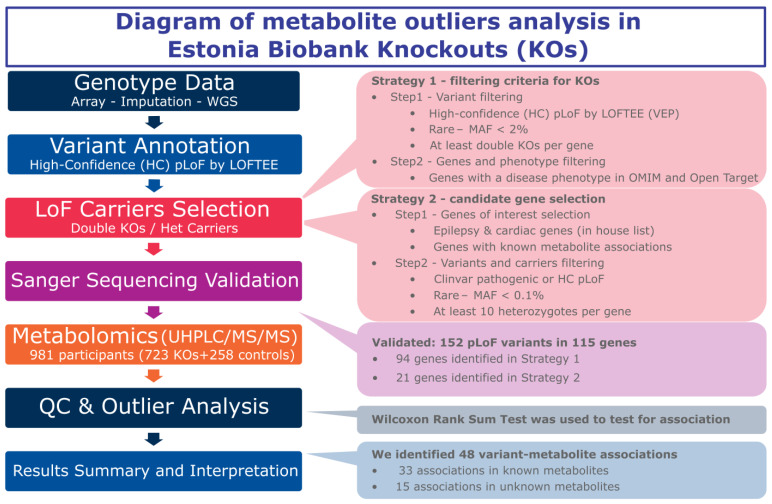
Study design and analysis workflow, including methods and summary results.

Strategy 2: We selected genes previously associated with epilepsy, cardiac conditions, and other diseases, as well as genes with known metabolite associations [4]. We included genes that have at least 10 heterozygous carriers of rare (MAF ≤ 0.1%) pathogenic or HC pLoF variants (Figure 1).

Genes without known disease associations were not considered in this analysis, as our focus was on genes already known to cause a condition.

The individual-level genotype of the selected variants of both the homozygous and heterozygous carriers were further validated by Sanger sequencing prior to analysis. We validated 585 KOs out of 628 selected by the first strategy (93% successful validation) and 138 heterozygous carriers out of 154 selected by the second strategy (90% successful validation) (Table S1). We were also able to validate 5 compound heterozygotes out of 20 candidates. A total of 729 EstBB genotypes of interest were successfully validated for 152 variants in 115 genes (Table S2). The number of carriers for each variant range from 2 to 12. A total of 6 participants were excluded because they had withdrawn their consent to use their data in research, reducing the total number to 723 participants. After validation by Sanger sequencing, the samples were sent for metabolomic profiling.

### 2.3. Metabolomics Profiling and Data Processing

Untargeted metabolomics profiling was conducted on ethylenediaminetetraacetic acid (EDTA) serum of 981 participants including 723 knockout samples identified in the previous step and 258 control samples who do not carry pathogenic or pLoF mutations in the selected genes. The detailed demographic characteristics of the study samples are summarized in Table S3. The profiling was conducted by Metabolon Inc. using the HD4 liquid chromatography–mass spectrometry (LC-MS) platform [17], followed by quality control and curation processing of the mass-spectrometry peak area data including batch-normalization.

A total of 1505 metabolites were quantified by the Metabolon HD4 platform, including 1179 chemically identified metabolites covering 9 super pathways (i.e., amino acids, carbohydrates, cofactors and vitamins, energy, lipids, nucleotides, partially characterized molecules, peptides, and xenobiotics) and 326 unnamed metabolites with unknown chemical structures (Table S4). Metabolites that were missing in more than 95% of the samples were excluded from the analysis (Figure S1). This exclusion criterion ensured that only reliably detected metabolites were included, resulting in a total of 1387 metabolites being analyzed in the present study (Table S4). Following the guidance provided by Metabolon, we did not perform imputation for missing metabolite values, as these are assumed to reflect concentrations below the limit of detection (LOD) of the LC-MS platform. Association tests were performed using unimputed data, excluding samples with missing values for each metabolite.

We performed the log transformation on the metabolites to reduce deviation from normality and then applied a two-stage approach for covariate adjustment. First, the log-transformed metabolite levels were regressed out of age, gender, and the first 10 genotype principal components (PCs). We tested for associations between the residuals from the first stage and covariates including body mass index (BMI), smoking status, education level, and sampling season, respectively. Simple linear regression was applied on BMI, one-way ANOVA was applied on other categorical covariates, and a threshold of *p* < 0.05 was used for determining significance. Due to the high-level of missingness in these covariates, in the second stage, we customized the regression model for each (log-transformed) metabolite by including age, gender, the first 10 PCs, and significantly associated covariates only, to preserve the sample. The normalized residual metabolite values were used for the downstream association analyses.

### 2.4. Variant-Metabolite Association Tests

We investigated the variant-metabolite association by directly comparing the metabolite levels between control samples and knockout samples for the gene of interest. We expected that a positive variant-metabolite association would be reflected by a significant difference in metabolite levels between the two groups. Significance was tested using a Wilcoxon rank sum test for each variant-metabolite pair.

Principal component analysis (PCA) showed that the first 368 PCs explained 90% variation in the normalized residual metabolite values. Therefore, to keep the study-wise type I error under 5%, we used a significance threshold of *p* < 0.05/368/152 = 9 × 10^−7^ to adjust for multiple tests on 1387 metabolites and 152 variants. The null hypothesis being tested here is that the knockout group and the control group have the same distribution with the same median. A location shift between the two distributions is expected if the variant is associated with the metabolite.

The power of the Wilcoxon rank sum test was estimated by simulation analysis. Simulated residual metabolite values were generated following normal distributions *N*(0,1) for control samples and *N*(β,1) for knockout samples. With the number of control samples fixed at 258, we simulated the dataset under different β values ranging from 0.5 to 5, and different knockout sample sizes ranging from 1 to 10. The power under each scenario was estimated by the proportion of positive tests among 10,000 trials. The statistical power of the test was evaluated by simulations where we generated the residual metabolite levels of 258 control samples and 1 to 10 knockout samples with different effect sizes. As shown in Figure S2, with the knockout sample size ≥ 5, when the effect size β ≥ 3, i.e., the location shift is greater or equal to 3 standard deviations, we can identify the association with ≥80% power and <5% study-wise Type I error. A smaller sample size may require a higher effect size for the association to be detected.

### 2.5. Variant-Disease Association Tests

The association between genes and clinical outcomes was tested for 981 samples.

Clinical diagnoses which appeared at least twice in the participant’s medical history were collected and aggregated according to the category represented by the first three digits of the ICD-10 code. Diagnosis categories with <5 cases and genes with <2 knockout samples were excluded from this analysis. A total of 588 diagnosis categories and 123 genetic variants were available for analysis after quality control.

Samples were stratified by gender and age group, and a conditional exact test given the strata margins was performed on each pair of gene and diagnosis category. The conditional maximum likelihood estimates of common odds ratio of having a diagnosis between knockout samples and control samples across strata was calculated. We used a significance threshold of *p* < 0.05/588/123 = 7 × 10^−7^ to adjust for multiple tests on 588 diagnosis and 123 variants.

## 3. Results

### 3.1. Identification of Variant-Metabolite Associations

We identified 33 variant-metabolite association signals in known metabolites (Table 1, Figure S3), and 15 association signals in unnamed metabolites (Table S5). Most of the associations (93%) were identified in genes selected by the first strategy. Out of the total 48 associations, 27 (56%) were found in genes that cause inborn errors of metabolism and 26 (54%) of reported associations were in gene targets of approved drugs or bioactive drug-like compounds. We were able to replicate the association between rs3918290 in *DPYD* and uracil (*p* = 2.34 × 10^−18^) [3] with a much smaller sample size. Additionally, we confirmed associations between *AGXT2* and 3-aminoisobutyrate (*p* = 1.61 × 10^−15^) [3,5,7], *UPB1* and 3-aminoisobutyrate (*p* = 2.25 × 10^−12^) [6], *ABCG5* and campesterol (*p* = 1.12 × 10^−8^) [4,6], *ACAD11* and X-24309 (*p* = 3.49 × 10^−7^) [5], and *FGGY* and both arabitol/xylitol and ribitol (*p* = 3.41 × 10^−7^) [6]. The remaining variant-metabolite associations were not previously reported in the literature, though the genes were all found associated with other metabolites [4,5,6].

### 3.2. Insights into Pyrimidine Degradation Pathway

**Figure 2:**
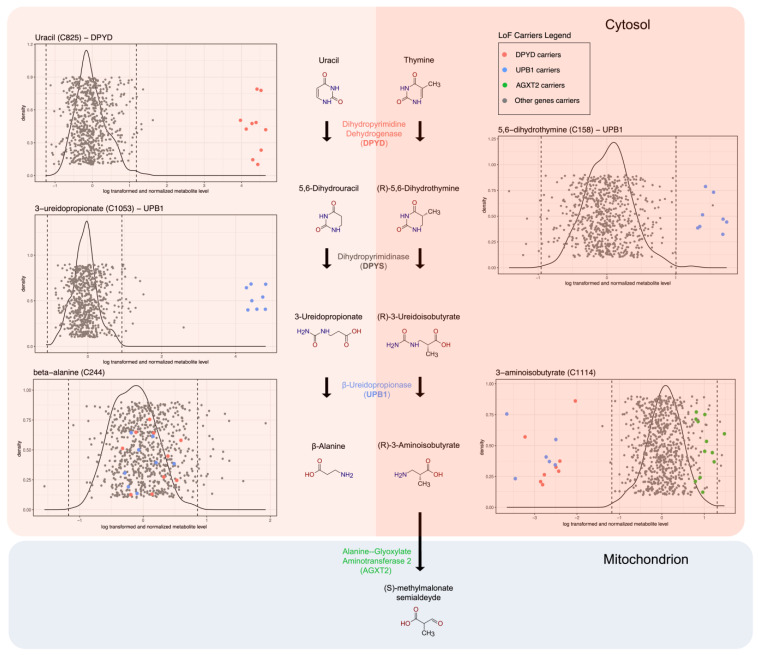
Pyrimidine degradation pathway and metabolism of β-aminoisobutyric acid (BAIBA): pyrimidines degradation pathway showing the genes associated with metabolites and the position of pLoF carriers compared to control distribution. BAIBA is produced in the cytosol through the thymine breakdown pathway and is further metabolized in mitochondria by alanine:glyoxylate aminotransferase 2 (AGXT2), BAIBA distribution of controls is plotted against the pLoF carriers for *DPYD* and *UPB1* genes that are part of pyrimidine degradation pathway, and AGXT2.

The top signals identified in our analysis are within the degradation pathway of the pyrimidine bases uracil and thymine (Figure 2), which involves three different enzymes—dihydropyrimidine dehydrogenase (encoded by *DPYD*), dihydropyrimidinase (encoded by *DPYS*, not selected in our analysis due to lack of knockout samples), and β-ureidopropionase (encoded by *UPB1*).

Dihydropyrimidine dehydrogenase, encoded by gene *DPYD*, is the initial and rate-limiting enzyme that catalyzes the reduction of uracil and thymine to 5,6-dihydrouracil and 5,6-dihydrothymine. Knockout samples of *DPYD* display a significantly higher level of uracil than control samples (*p* = 2.34 × 10^−18^), as well as knockout samples of other genes (Figure 2). In contrast, 5,6-dihydrouracil—the direct product of DPYD-mediated uracil reduction—was entirely missing in all *DPYD* knockout samples, while it was detected in the majority of other samples (missing in 61 out of 971). We interpreted this complete missingness as likely reflecting concentrations below the LC–MS detection limit. Supporting this, a Fisher’s exact test confirmed a significantly higher missingness rate in knockouts (*p* = 2 × 10^−12^). Together with elevated uracil levels, this pattern supports a pathway-level effect of *DPYD* loss consistent with reduced function of dihydropyrimidine dehydrogenase. However, we acknowledge that technical artifacts cannot be fully excluded as alternative explanations of these observations. We did not observe any significant difference in the levels of thymine and 5,6-dihydrothymine between *DPYD* KOs and other samples.

β-ureidopropionase, encoded by *UPB1*, catalyzes the third step in the thymine degradation pathway in which 3-ureidopropionate and 3-ureidoisobutyrate are converted to β-alanine and 3-aminoisobutyrate. We detected significant associations between *UPB1* and 3-ureidopropionate (*p* = 3.73 × 10^−15^), and between *UPB1* and 3-aminoisobutyrate (*p* = 2.25 × 10^−12^). Though 3-ureidopropionate was not measured in our study, we detected a strong association (*p* = 1.79 × 10^−15^) between *UPB1* and 5,6-dihydrothymine, which is the reactant of the reaction generating 3-ureidopropionate in the second step in the pathway. We observed that *UPB1* KOs have an elevated level of 3-ureidopropionate and 5,6-dihydrothymine, and a reduced level of 3-aminoisobutyrate, which is consistent with the flow of the degradation pathway.

### 3.3. Identification of Variant-Disease Associations

The analysis we performed testing the association between genes and clinical outcomes did not produce any significant results at the significance Bonferroni threshold of *p* ≤ 7 × 10^−7^. In Table S6, we reported all associations with a nominal significant threshold of *p* ≤ 0.05.

To complement to this approach, we looked up the EHR of the pyrimidine pathway gene KOs and showed that the closest matches among ICD-10 codes are “Other disorders of purine and pyrimidine metabolism (E79.8)” and “Disorder of purine and pyrimidine metabolism, unspecified (E79.9)”, confirming that the KOs have been diagnosed with disorders of purine and pyrimidine metabolism.

## 4. Discussion

Our study builds on Saleheen et al.’s work [8] on natural human KOs in a Pakistani cohort, which focused on biochemical and disease traits. We expanded this research by examining individual KOs metabolomes, specifically in HC pLoF variants validated by Sanger sequencing.

We performed untargeted metabolomics on a subset of the EstBB to investigate the correlation between pLoF genetic variants and metabolite levels in human KOs. By analyzing human gene knockouts within the EstBB, we aim to elucidate gene functions, disease mechanisms, and potential therapeutic targets. This methodology is particularly valuable for understanding the impact of rare pLoF variants, which are frequently underrepresented in population-based studies [18,19]. Ultimately, we identified 48 variant-metabolite associations involving 22 genes and 43 metabolites. Interestingly, 27 (56%) of these associations involved genes known to cause inborn errors of metabolism. For instance, knockouts in the *DPYD* gene were associated with elevated levels of uracil, corroborating the role of DPD deficiency in severe 5-Fluorouracil toxicity. Additionally, significant changes in metabolite levels were observed in genes involved in the pyrimidine degradation pathway, such as *DPYD* and *UPB1*. Furthermore, 14 (29%) of the identified associations were in genes mapped to metabolite loci (metabolite GWASs). These findings underscore the relevance of our study in elucidating the impact of KOs in gene identified through both rare and common genetic variants.

A recent study by Aziz Belkadi et al. [20] in a Qatari cohort focused on protein-changing variants, including missense variants. They reported associations with metabolite for genes like *AGXT2*, *ABCG5*, and *UPB1*. *AGXT2*/3-aminoisobutyrate association was reported with the same variant (rs114286107) as in our study. However, for *UPB1*/3-ureidopropionate, we identified additional metabolites (3-aminoisobutyrate and 5,6-dihydrothymine) in a different variant, enhancing our understanding of the pyrimidine pathway, and we identified *ABCG5*/campestrol in a pathogenic pLoF variant, while they found it in missense mutations. Belkadi et al. [20] metabolite associations analysis was restricted to homozygotes ranked in the top 20 or bottom 20 for metabolite levels and used permutations for estimating null distribution and determining FDR. We employed the Wilcoxon rank sum test, providing a different methodological approach. Our stricter filtering and unique cohort, characterized by high homozygosity due to recent historical bottlenecks, allowed us to identify associations not reported in their paper [21].

Most of the identified associations had clear biochemical functions described in the literature, allowing us to better elucidate gene functions and their implications in disease. A prime example involves genes and metabolites in the pyrimidine degradation pathway. We found that two out of the three genes in this pathway (*DPYD* and *UPB1*) had at least two knockouts (KOs) in the EstBB. These genes were associated with four out of the eight measured metabolites within the pathway, providing opportunities to further investigate the biochemistry, gene function, and phenotypic consequences.

Our findings align with and expand upon previous research on the pyrimidine degradation pathway in several ways. First, the association of *DPYD* knockouts with elevated uracil levels supports existing knowledge about dihydropyrimidine dehydrogenase (DPD) deficiency and its clinical implications.

Mutations in *DPYD* and *UPB1* can lead to deficiencies in DPD and beta-ureidopropionase, respectively, causing severe neurological issues and other related symptoms. [22,23]. Our study found that 10 *DPYD* KOs had significantly elevated uracil levels, potentially due to chemotherapy treatment. In fact, the use of fluoropyrimidine in cancer chemotherapy, such as 5-fluorouracil and capecitabine, can exacerbate DPD deficiency, causing severe toxicity in 10–40% of patients [24]. Additionally, the *DPYD* KOs were identified in the rs3918290 splice donor variant, which is utilized to guide chemotherapy dosing. Notably, 5,6-dihydrouracil was missing in all *DPYD* KO samples, with a significantly higher missingness rate compared to other samples. While LC–MS-based metabolomics data can be affected by non-biological factors such as compound instability, ion suppression, or batch effects, several observations support a biological interpretation in this case: uracil, the upstream metabolite, was significantly elevated in the same knockout samples, and statistical testing confirmed that the missingness of 5,6-dihydrouracil was strongly associated with *DPYD* loss. These findings, viewed in the context of the pathway, are consistent with reduced dihydropyrimidine dehydrogenase activity due to *DPYD* loss-of-function. Nevertheless, we acknowledge that technical artifacts cannot be fully ruled out and recommend further validation. In beta-ureidopropionase deficiency, patients accumulate specific metabolites such as of N-carbamyl-β-aminoisobutyric acid, N-carbamyl-β-alanine (also known as 3-ureidopropanoate), uracil, thymine, 5,6-dihydrouracil, and 5,6-dihydrothymine in urine and plasma, leading to variable neurological phenotypes (from asymptomatic to developmental disorder) [25]. We observed the splice acceptor variant rs143493067 in *UPB1* in eight KOs, all showing increased levels of 5,6-dihydrothymine and N-carbamyl-β-alanine, adding further evidence to the pathogenicity of the rs143493067 variant through the disruption of RNA splicing, which has been reported with conflicting classifications in ClinVar [26].

We also found that six KOs in *UPB1* (rs143493067) and eight KOs in *DPYD* (rs3918290) had a significant decrease in β-aminoisobutyric acid (BAIBA) levels, the final product of thymine degradation. This decrease in BAIBA levels could impact the activation of the glycine receptor and the efficacy of γ-aminobutyric acid, potentially affecting neurological functions [22].

Furthermore, we observed an increase in BAIBA levels in 12 KOs in *AGXT2* (rs114286107), a gene not directly linked to the pyrimidine degradation pathway but previously associated with BAIBA metabolism in in vivo and in vitro study [27,28] and in metabolite GWAS in urine [29], showing that BAIBA is metabolized by alanine:glyoxylate aminotransferase 2 (*AGXT2*). Those studies suggest that in humans, BAIBA is mainly metabolized by *AGXT2* and rs37369 is associated with a higher urinary excretion of BAIBA (hyper-BAIB aciduria), a heritable trait which first was described in 1951 [30].

*AGXT2* has several other substrates besides BAIBA, including SDMA. Plasma SDMA levels have been reported to correlate with serum creatinine level in individuals with impaired renal function that is, in turn, a risk factor for cardiovascular events and total mortality. The mechanism through which SDMA increased risk for total mortality is not well understood, but increased levels of BAIBA in *AGXT2* KOs could have further implications in disease progression [27]. However, another study presented a potentially protective effect of BAIBA on atherosclerosis, cancelling out the risk associated with elevated levels of SDMA in individuals with the *AGXT2* defect [31].

Furthermore, BAIBA is reported to have an effect on respiratory function of human podocyte and renal function [32] as well as human metabolism benefitting body fat mass, plasma lipoproteins levels, insulin sensitivity, inflammatory responses, and possibly for the arterial wall [31].

We found Acyl-CoA Dehydrogenase Family Member 11 (*ACAD11*) to be associated with an unknown metabolite reported previously [5] as well as six other known metabolites, three amino fatty acids (2-aminooctanoate, N-acetyl-2-aminooctanoate, and 2-aminoheptanoate), two monohydroxy fatty acids (2-hydroxylaurate and 2-hydroxyoctanoate), and an amino acid (2-ketocaprylate) not previously reported. *ACAD11* participates in beta-oxidation and energy production but could also play a role in the metabolism of specific fatty acids to control fatty acids composition of cellular lipids in brain. rs41272317 is a splice donor variant that is rare in gnomAD v4 (MAF = 0.002%) and is not reported in Clinvar. Additionally, *ACAD11* has been identified as a metabolic target of the p53 protein, which is crucial for cell survival under metabolic stress. p53 activates *ACAD11* to enhance fatty acid metabolism, supporting oxidative phosphorylation (OXPHOS) and managing reactive oxygen species (ROS) levels. This pro-survival function of p53, including its ability to limit ROS accumulation, depends on its activation of genes like *ACAD11*. Targeting *ACAD11* or other p53-regulated pathways could potentially create vulnerabilities in tumor cells that rely on these mechanisms for survival. This connection underscores the importance of *ACAD11* in the broader context of p53′s role in cellular metabolism and survival, offering potential avenues for therapeutic interventions in cancer treatment [33].

Our approach, which leverages a population with a higher proportion of homozygous pLoF carriers [21], suggested to be a cost-effective and powerful method for detecting large-effect genetic variants. This complements traditional population-based methods and offers a new avenue for studying rare variants.

It is important to mention that the main limitation of this study is the reduced statistical power to detect associations with the current sample size and the absence of validation in an independent cohort. Also, although we validated all pLoF variants using Sanger sequencing, we do not have any proof for the loss of gene function. Future research should aim to validate these associations through functional experiments and explore their clinical implications further.

## 5. Conclusions

Overall, our findings contribute to the growing body of knowledge on the biochemical consequences of genetic variation integrating metabolomics with genetic data. This work enhances our understanding of gene function and its phenotypic consequences, ultimately aiding in drug discovery and the development of targeted therapies.

## Figures and Tables

**Table 1:** Variant-metabolite associations detected by Wilcoxon rank sum one-sided test (chemically identified metabolites with known pathway); in bold novel associations; * indicates the gene is a known drug targets and ** indicates the gene is reported in OMIM.

Gene Name	Variant ID	Metabolite Name	# of Controls	# of Carriers	# of Carriers 3 SD Outlier	Direction	*p*-Value	Drug	OMIM Disorder Associated with Gene	Strategy
ACAD11 *	rs41272317	2-aminoheptanoate	257	12	1	+	5.94 × 10^−9^	bioactive compound	-	1
ACAD11 *	rs41272317	2-aminooctanoate	196	11	3	+	2.15 × 10^−14^	bioactive compound	-	1
**ACAD11** *	**rs41272317**	**2-hydroxylaurate**	**254**	**11**	**1**	**+**	**7.45 × 10** ^**−7**^	**bioactive compound**	**-**	**1**
ACAD11 *	rs41272317	2-hydroxyoctanoate	257	12	2	+	1.32 × 10^−11^	bioactive compound	-	1
ACAD11 *	rs41272317	2-ketocaprylate	257	12	0	+	1.43 × 10^−9^	bioactive compound	-	1
AGXT2 **	rs114286107	3-aminoisobutyrate	258	12	1	+	1.61 × 10^−15^	-	urinary excretion of beta-aminoisobutyric acid	1
DPYD *^,^**	rs3918290	3-aminoisobutyrate	258	8	8	-	1.79 × 10^−15^	Phase III	dihydropyrimidine dehydrogenase deficiency; 5-fluorouracil toxicity	1
**UPB1** **	**rs143493067**	**3-aminoisobutyrate**	**258**	**6**	**6**	**-**	**2.25 × 10^−12^**	**-**	**beta-ureidopropionase deficiency**	**1**
**PDE11A** *^,^**	**rs781747963**	**3-hydroxybutyrate (BHBA)**	**258**	**10**	**0**	**-**	**1.47 × 10^−7^**	**bioactive compound**	**pigmented nodular adrenocortical disease, primary, 2**	**1**
**A2ML1** **	**rs202067416**	**3-hydroxylaurate**	**258**	**12**	**1**	**-**	**3.66 × 10^−7^**	**-**	**susceptibility to otitis media**	**1**
**PTH2R** *	**rs61742329**	**3-ureidopropionate**	**235**	**12**	**0**	**+**	**6.52 × 10^−7^**	**bioactive compound**	**-**	**1**
**UPB1** **	**rs143493067**	**3-ureidopropionate**	**235**	**8**	**8**	**+**	**3.73 × 10^−15^**	**-**	**beta-ureidopropionase deficiency**	**1**
**UPB1** **	**rs143493067**	**5,6-dihydrothymine**	**258**	**8**	**8**	**+**	**1.79 × 10^−15^**	**-**	**beta-ureidopropionase deficiency**	**1**
FGGY *	rs41287704	arabitol/xylitol	258	3	3	+	3.41 × 10^−7^	bioactive compound	-	1
**COL23A1**	**rs2973744**	**asparagine**	**204**	**3**	**2**	**+**	**6.86 × 10^−7^**	**-**	**-**	**1**
**PTH2R** *	**rs61742329**	branched-chain, straight-chain, or cyclopropyl 12:1 fatty acid *	**258**	**12**	**1**	**-**	**4.36 × 10^−7^**	**bioactive compound**	**-**	**1**
ABCG5 *^,^**	rs199689137	campesterol	138	8	1	+	1.12 × 10^−8^	bioactive compound	sitosterolemia 2	2
**NPC2** **	**rs140130028**	**cysteine s-sulfate**	**258**	**11**	**2**	**+**	**2.15 × 10^−10^**	**-**	**Niemann-pick disease, type C2**	**1**
**MPO** *^,^**	**rs35897051**	**fructose**	**258**	**10**	**3**	**+**	**3.58 × 10^−7^**	**Phase III**	**myeloperoxidase deficiency; susceptibility to Alzheimer disease**	**1**
**OBSL1** **	**rs140825693**	**gamma-glutamylphenylalanine**	**257**	**11**	**0**	**+**	**7.86 × 10^−8^**	**-**	**3-M syndrome 2**	**1**
**OBSL1** **	**rs140825693**	**gamma-glutamyltyrosine**	**257**	**11**	**1**	**+**	**6.75 × 10^−8^**	**-**	**3-M syndrome 2**	**1**
**CFHR3** **	**rs138839071**	**glycochenodeoxycholate**	**253**	**10**	**0**	**+**	**4.34 × 10^−7^**	**-**	**susceptibility to the development of atypical hemolytic uremic syndrome-1**	**1**
**CFHR3** **	**rs138839071**	**glycocholate**	**258**	**11**	**0**	**+**	**2.37 × 10^−7^**	**-**	**susceptibility to the development of atypical hemolytic uremic syndrome-1**	**1**
**A2ML1** **	**rs202067416**	**isoleucine**	**257**	**11**	**1**	**+**	**2.00 × 10^−8^**	**-**	**susceptibility to otitis media**	**1**
**A2ML1** **	**rs202067416**	**leucine**	**257**	**11**	**1**	**+**	**1.02 × 10^−7^**	**-**	**susceptibility to otitis media**	**1**
**OBSL1** **	**rs140825693**	**methionine**	**205**	**8**	**1**	**+**	**6.57 × 10^−7^**	**-**	**3-M syndrome 2**	**1**
ACAD11 *	rs41272317	N-acetyl-2-aminooctanoate *	257	12	3	+	2.32 × 10^−12^	bioactive compound	-	1
**NPC2** **	**rs140130028**	**ornithine**	**258**	**11**	**0**	**+**	**8.95 × 10^−11^**	**-**	**Niemann-pick disease, type C2**	**1**
**A2ML1** **	**rs202067416**	**pyrraline**	**249**	**11**	**1**	**+**	**5.19 × 10^−7^**	**-**	**susceptibility to otitis media**	**1**
FGGY *	rs41287704	ribitol	255	3	3	+	3.53 × 10^−7^	bioactive compound	-	1
**CLCN1** **	**rs55960271**	**taurine**	**258**	**12**	**1**	**+**	**1.46 × 10^−7^**	**-**	**myotonia congenita**	**1**
DPYD *^,^**	rs3918290	uracil	257	10	10	+	2.34 × 10^−18^	Phase III	dihydropyrimidine dehydrogenase deficiency; 5-fluorouracil toxicity	1
**A2ML1** **	**rs202067416**	**vanillic acid glycine**	**230**	**10**	**0**	**+**	**3.84 × 10^−7^**	**-**	**susceptibility to otitis media**	**1**

## Data Availability

EstBB data cannot be published in repositories and information on data access can be found at https://genomics.ut.ee/en/content/estonian-biobank (accessed on 8 May 2025).

## Supplementary Materials

The following supporting information can be downloaded at: https://www.mdpi.com/article/10.3390/metabo15050323/s1, Table S1: Number of genotypes selected by strategy and validation success, Table S2: list of genes and variants validated by Sanger sequencing, Table S3: Characteristics of 981 Estonian participants included in the analysis, Table S4: Metabolite distribution in Super pathways, Table S5: Variant-Metabolite Associations detected by Wilcoxon Rank Sum One-Sided Test (Metabolites with Unknown Pathway), Table S6: Variant-ICD Associations detected by Conditional Exact Test, Figure S1: Proportion of samples with missing metabolite levels, Figure S2: Power Analysis for Wilcoxon Rank Sum Test, Figure S3: STRING network analysis, including all known metabolites and genes found significantly associated.
