# Supplementary figures and images for: Plasma Metabolic Outliers Identified in Estonian Human Knockouts
Source: Metabolites. 2025 May 13;15(5):323. doi: 10.3390/metabo15050323 (PMC12114030; doi:10.3390/metabo15050323)

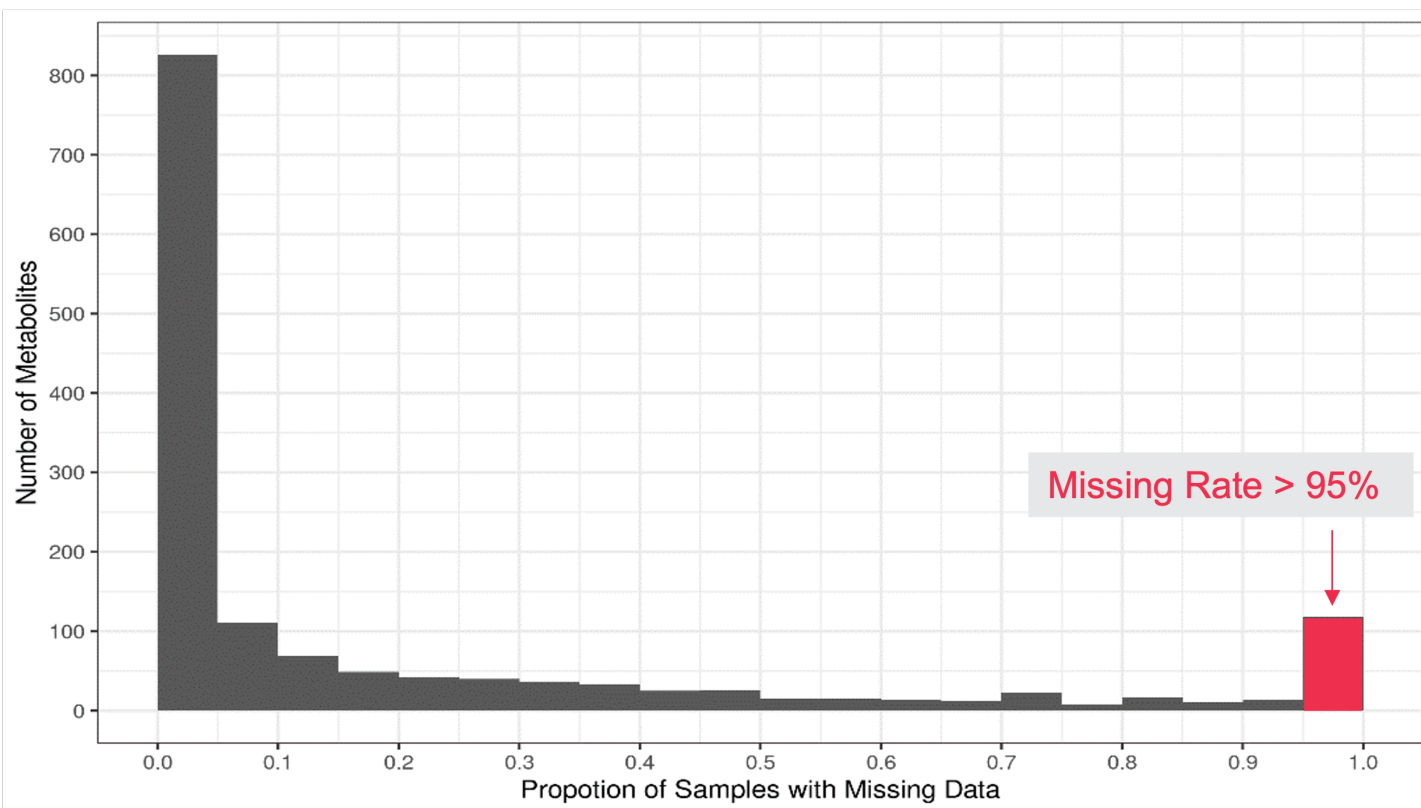

Supplement: Supplementary file 1 [file metabolites-15-00323-s001.zip › FigureS1.pdf]

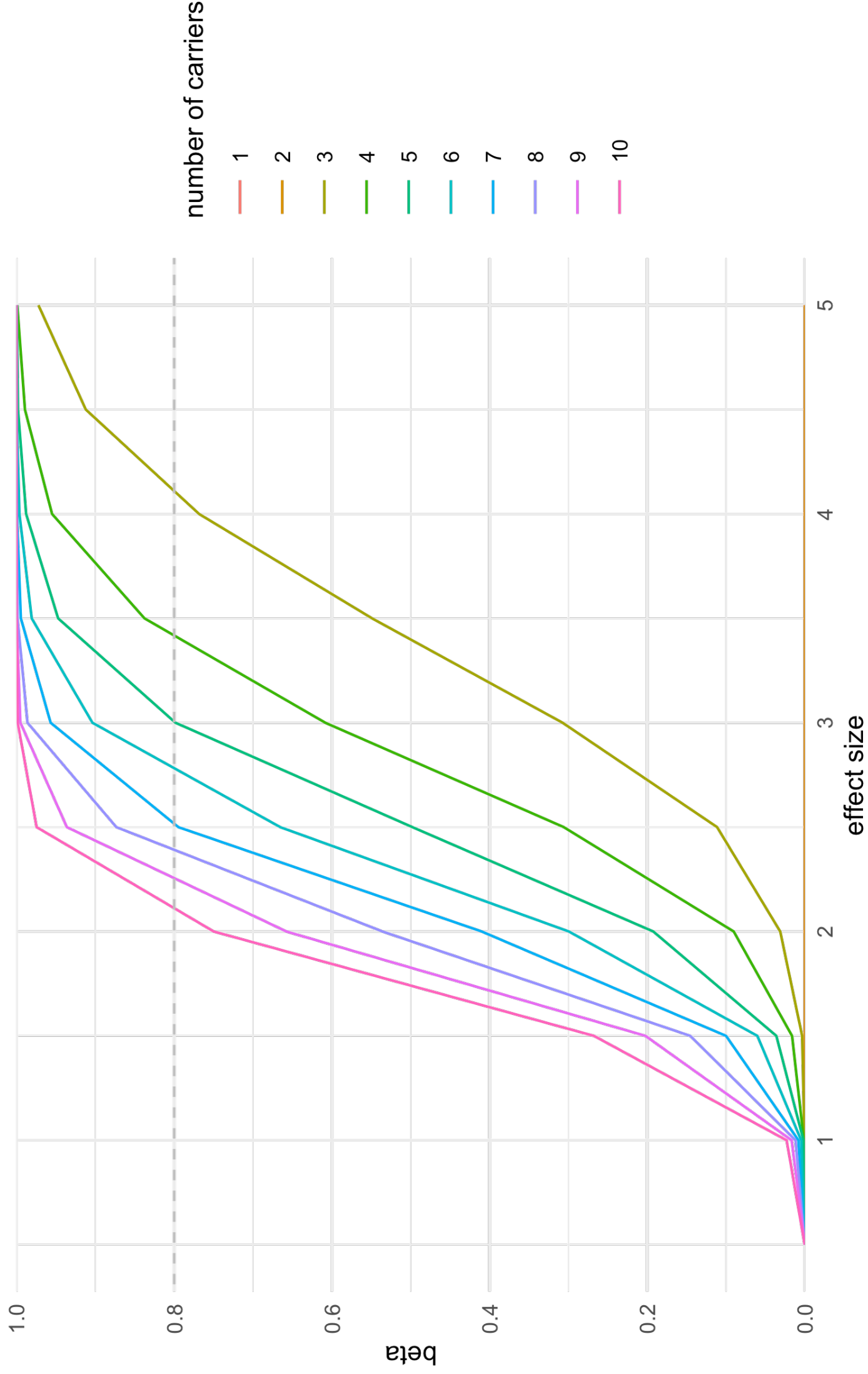

Supplement: Supplementary file 1 [file metabolites-15-00323-s001.zip › FigureS2.pdf]

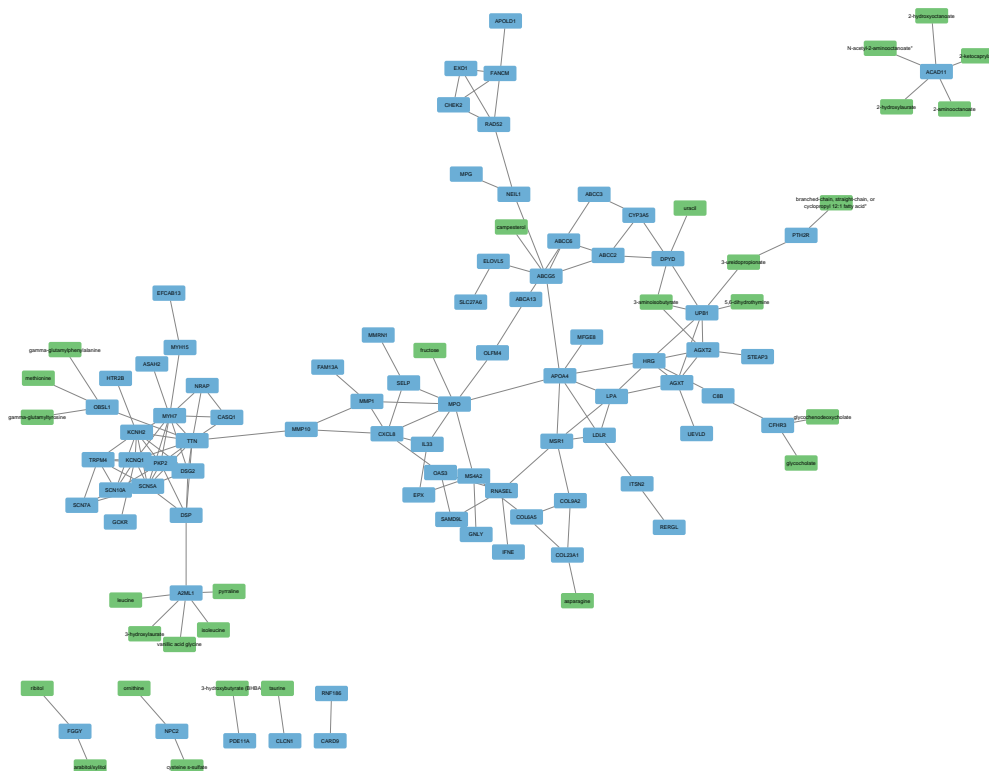

Supplement: Supplementary file 1 [file metabolites-15-00323-s001.zip › FigureS3.pdf]
